# Proceedings of Oneida County Medical Society

**Published:** 1889-06

**Authors:** 


### PROCEEDINGS OF THE ONEIDA COUNTY HOMŒOPATHIC MEDICAL SOCIETY.

The quarterly meeting of the Oneida County Homœopathic Medical Society was held April 16th, in Rome (N. Y.) Present, Drs. S. O. and N. C. Scudder, Southwick, Touseley, and Gifford, Rome; Brainard, of Little Falls, and Wells, of Utica.

In the absence of the President and Secretary, Dr. S. O. Scudder was elected President and L. B. Wells Secretary *pro tem.*

The Secretary read the letter of Judge Barrett to the *New York Medical Times* in relation to medical practice, which was fully discussed by all present.

Dr. L. B. Wells, of Utica, read the following:

"The answer (to a question by the editor of the *New York Medical Times*) if correct, has an important bearing upon all practitioners of every school of practice. The principal extracts will be found in the *New York Medical Times* for April 1st. This opinion has a direct bearing upon the organization of the homœopathic medical societies as to who shall be its members. In answer to questions of this nature, some few years since, Judge Davis, an eminent jurist of New York city, gave his opinion. He quotes the laws of this State in regard to the organization of homœopathic county medical societies. Homœopathic physicians, not less than five, may assemble at the county seat and organize a county society. He asks the question, 'Who are homœopathic physicians?' His answer is, 'Those who practice Homœopathy according to the law of cure as promulgated by Hahnemann.' He says 'Not allopaths, not eclectics, not electricians, not hydropathists.'

"The County Homœopathic Medical Society of New York County was organized on the principles of pure Homœopathy, and after the opinion of Judge Davis was published, a large number of members who were eclectic in practice withdrew from the society. Our county society was organized on the same platform of pure Homœopathy. Our first State society was organized on the pure platform. In the transactions of the State society of 1872 you will find the Constitution and By-Laws of that and the Constitution and By-Laws of the new society. In the first you will perceive that the law *similia similibus curantur* was recognized, but not as published by the new, while Dr. H. M. Paine was Secretary. But let us now refer to the facts that according to reports to boards of health those who practice pure Homœopathy have the most favorable results, showing the least mortality.

"Judge Barrett's opinion leaves the impression that homœopathic treatment often fails, making resort to other means necessary by the knowledge and consent of the patient. This is an erroneous conclusion. The homœopathic physician who carefully and thoroughly studies the Homœopathic Materia Medica, the result of provings upon the healthy organism, and the characteristics of drugs will no more resort to Quinine, Morphine, Bromide of Potash, Chloral, etc., as used by our allopathic brethren of the old-school practice than the skillful, keen-eyed rifleman would to the old-fashioned, long-time-discarded blunderbuss. The failure is not, therefore, of Homœopathy but the practitioner. From this cause alone Homœopathy has suffered more injury from wounds in the house of its professed friends than from all the opposition and ridicule of its foes."

He also read the following written by Dr. I. Dever, of Clinton:

"The Utica *Morning Herald*, of April 3d, contains an extract from the *New York Medical Times* which is intended to mislead the public in regard to the different medical practices. Judge Barrett is quoted as authority, and made to voice the opinion of the *Medical Times*. We do not take exceptions to the legal aspect of the question, but when Judge Barrett gives his opinion in regard to the relative merits of the different schools of medicine it is but the opinion of a layman, and not that of a learned jurist, as the *Times* would have it, from the fact that the evidence is not all in, and he is ruling in favor of the *Medical Times* without evidence. He presupposes a case in which the homœopath is made to fail with his remedies and fall back to allopathic drugging and blind stabbing, a hypothesis upon which the *Medical Times* and Judge Barrett base an opinion and rule against Homœopathy. But suppose we present a small amount of evidence from the other side? During the epidemic of 1849 and 1850 Drs. Pulte and Ehrman, of Cincinnati, Ohio, treated cholera with a loss of three per cent., while the regular practice lost fifty per cent. Dr. Rubini, of Naples, Italy, treated seven hundred patients in 1854 with a loss of only three. But we will see how Homœopathy succeeds in the dreaded disease of diphtheria. During an epidemic of diphtheria in Philadelphia, Dr. Neidhard (a homœopath) lost but two patients out of three hundred.

"Dr. J. P. Dake, of Pittsburg, Pa., lost seven out of one hundred and ninety-three cases treated. Dr. J. N. Brigham, of Grand Rapids, Mich., lost one case out of fifty treated during the summer of 1887. Out of two hundred and fifty cases treated by Dr. DeForest Hunt, of Grand Rapids, Mich., during the summer of 1887, he lost eight patients. The average death rate in later years, under old-school treatment, is about twenty per cent. The *Medical Times* and its friends would create an examining board, fabricate a case against Homœopathy, and condemn all practitioners of that school without a hearing.

"Clinton, N. Y., April 4th, 1889."

The question of using allopathic measures was called up and was ventilated. Dr. N. C. Scudder thought the regulation of doses should be according to the judgment of the physician to suit the constitution and temperament of the patient. Dr. Wells thought there was nothing in the system of Homœopathy to forbid the use of large doses of medicine—allopathy doses, so-called —if they should be deemed desirable in any case.

Dr. S. O. Scudder said that the law of *similia similibus curantur* was a very important discovery, and that the practice should be confined within that circle. He spoke at some length on the theory.

Dr. Brainard said that he believed in the homœopathic law of applying medicine, but he believed the size of doses should be regulated by the judgment of the attending physician solely. He thought many of Hahnemann's ideas and theories were fallacious. He cited some instances of proof.

The subject of Psora and Hahnemann's theory of the same was freely discussed, with reports of cases where suppression of itch by external means resulted in various forms of chronic diseases. Various cases of hernia were reported, with a discussion of the best means of reducing the same to avoid the necessity of resorting to an operation, attended often with danger. Adjournment.

